# Work life of persons with asthma, rhinitis, and COPD: A study using a national, population-based sample

**DOI:** 10.1186/1745-6673-1-2

**Published:** 2006-02-02

**Authors:** Edward Yelin, Patricia Katz, John Balmes, Laura Trupin, Gillian Earnest, Mark Eisner, Paul Blanc

**Affiliations:** 1 Division of Rheumatology, Department of Medicine, University of California, San Francisco, San Francisco, CA 94143-0920, USA; 2 Institute for Health Policy Studies, University of California, San Francisco, San Francisco, CA 94143-0920, USA; 3 Division of Occupational and Environmental Medicine, Department of Medicine, University of California, San Francisco, San Francisco, CA 94143-0924, USA; 4 Cardiovascular Research Institute, University of California, San Francisco, San Francisco, CA 94143-0924, USA

## Abstract

**Objective:**

To estimate the duration of work life among persons reporting a physician's diagnosis of COPD, asthma, or rhinitis compared to those with select non-respiratory conditions or none and to delineate the factors associated with continuance of employment.

**Methods:**

Persons ages 55 to 75 reporting a physician's diagnosis of COPD, asthma, or rhinitis as well as those without any of these conditions were identified by random-digit dialing (RDD) in the continental U.S and administered a structured survey. We used Kaplan-Meier life table analysis to estimate the duration of work life among persons with and without the three conditions and Cox proportional hazard regression to examine the role of demographic and work characteristics in the proportion leaving employment in each time interval.

**Results:**

Persons with COPD, asthma, and rhinitis were no less likely than the remainder of the population to have ever worked, but those with COPD were less likely to be working when interviewed or as of age 65, whichever came first. As of age 55, only 62 percent of persons with COPD continued to work versus 72 and 78 percent of persons with asthma and rhinitis, respectively. Persons with COPD, asthma, and rhinitis all had an elevated risk of leaving work prior to age 65 relative to those without chronic conditions, with and without adjustment for demographic and work characteristics.

**Conclusion:**

COPD and to a lesser extent asthma and rhinitis were associated with a substantially shortened work life, an effect not due to demographic and work characteristics.

## Background

The impact of chronic respiratory conditions on employment even when work is not a cause of these conditions has been the subject of a growing literature. Most of the studies have concerned the impact of asthma [1-18], although a few concern other discrete respiratory conditions, including allergic rhinitis [14,19], cystic fibrosis [20], and chronic bronchitis [17].

The vast majority of studies have used clinical samples, with the attendant risk that the work disability rate will be overestimated since persons sampled in clinical environments are more likely to have severe disease [21]. Recently there have been several studies that use population-based sampling frames [2,10,14,16,17,22-24]. However, each of these studies have been limited in scope: conducted only on adults from a younger age group (20 to 44) [10,17], a single birth cohort [2], or a single state or region of a state [14,23]; using data sources with limited employment data [24]; measuring only the parental employment effects of childhood asthma [16]; or focusing on extent of acute work "impairment" days rather than on such measures of chronic impact as actual employment status [22].

In addition, none of the foregoing population-based studies have attempted to estimate the impact of respiratory conditions on the duration of work life, a critical dimension of the impact of illness because premature withdrawal from the labor market affects the magnitude of retirement benefits (including both private pensions and Social Security) and the assets accrued which one may drawn on in retirement [25]. Moreover, persons withdrawing from employment prior to age 65 may not have health insurance benefits until they reach age 65 [26].

The present study overcomes some of the limitations in the previous population-based studies of employment among persons with respiratory conditions by using a national sample, including persons with several respiratory conditions (and with none), encompassing those from an age range (55 to 75) when employment impacts are most likely to be manifest, and taking a complete work history of the respondents. As a result, we are able to estimate the duration of work life among persons from a population-based sample with COPD, rhinitis, and asthma compared to those with non-respiratory chronic conditions or no conditions. We can also examine the role of demographic characteristics and work-related factors at the longest job held in accounting for differences among the groups in the proportion leaving employment in each time interval.

## Methods

### Overview

We used random-digit dialing techniques to develop a population-based sample of persons reporting a physician's diagnosis of COPD, rhinitis, and asthma, non-respiratory chronic conditions, or no chronic conditions. These individuals were then administered a structured telephone survey about their medical conditions, demographic characteristics, and work history. Details about the survey methods of the study have been published previously [27]. The protocol for the study was approved by the Committee on Human Research of the University of California, San Francisco.

We used Kaplan-Meier life table analysis to estimate the duration of the work life of persons with each of the respiratory conditions and the groups with and without non-respiratory chronic conditions and Cox proportional hazards regression to estimate the demographic characteristics and work-related factors associated with the continuance of employment among the entire study sample.

### Sampling

The study population arose from three independent population-based subsamples derived from random-digit dialing interviews conducted in either English or Spanish between April and August, 2001. For each of the subsamples, a screening question was administered to identify eligible households with someone between 55 and 75 years of age; if two or more individuals in the household were in the age range, one was chosen at random.

The first subsample included 1,001 persons recruited from the 48 contiguous states of the U.S. The second and third subsamples were limited to specific geographic "hot spots," based on Health Service Areas with the highest COPD mortality rates, derived from the National Institute of Occupational Safety and Health Atlas of Respiratory Disease Mortality in the United States: 1982 – 1993 (20). For the second and third subsamples, we selected telephone area codes that closely corresponded to the areas in the top quartile of elevated age-adjusted mortality rates. The second subsample included 1,002 participants recruited through simple random sampling of these "hot spot" area codes. The third subsample included 110 respondents also selected from the "hot spot" areas, but excluding all individuals not reporting a physician diagnosis of one of three conditions subsumed within the overall rubric of COPD (chronic bronchitis, emphysema, or chronic obstructive lung disease) or asthma. The purpose of the second and especially the third subsamples was to enrich the overall sample for persons with these target conditions.

The resulting overall sample consisted of 2,113 individuals aged 55 to 75. Of these, 352 reported a physician diagnosis with one of the conditions within the overall rubric of COPD and 122 reported a physician diagnosis of asthma. In the course of administering the complete structured telephone survey to all 2,113 individuals, 194 reported a physician diagnosis of rhinitis or hay fever (hereafter, "rhinitis"). Small numbers of respondents indicated that they had a physician's diagnosis of sleep apnea (n = 53) or lung cancer (n = 8) in the absence of COPD, asthma, or rhinitis. Persons with sleep apnea or lung cancer in the absence of these other conditions were included in the overall rubric of respiratory disease, but were too few in number for reliable analysis as discrete conditions. In addition to those with respiratory conditions, 760 reported a physician diagnosis of one or more non-respiratory conditions in the absence of respiratory conditions from a brief checklist of conditions (diabetes, arthritis, congestive heart failure, and coronary artery disease or heart attack), and 632 reported no chronic conditions.

Some persons reported two or more respiratory conditions from among COPD, asthma, and rhinitis. Persons with COPD and another condition were classified as having COPD. Persons with asthma and rhinitis were classified as having asthma.

The overall rate of completion of the entire survey among households in which it could be determined that there were one or more persons 55 to 75 years of age was 53 percent. Among the 2,113 respondents, 2,005 (95 percent) had no missing data on any of the variables used in the analysis (see below). The remaining cases (5 percent) were eliminated from the analysis, but given the low frequency of missing data, this is unlikely to affect the results.

### Content of interview

All respondents completed identical structured telephone-surveys covering respiratory symptoms and medications and other treatments for those symptoms; health behaviors, including smoking history and current smoking status; overall health status, including the selected co-morbid conditions listed above and general health status as measured by the SF-12 instrument [28]; demographic characteristics and socioeconomic status; and employment history and current employment status. The health characteristics were reported for the time of the interview and, thus, could not be used as predictors of current employment status or time until withdrawal from employment since change in employment may have occurred prior to the worsening of health. We use the health characteristics for descriptive purposes only (see Results, below).

The employment section used established batteries [29] to collect information about the respondents' current employment status and the nature of their current or most recent job and the job held for the longest time during their careers. Items included were the number of years on the job, open-ended questions about occupation and industry – later coded to the U.S. 2000 Census codes [30], and self-reported exposure to vapors, gas, dust, and fumes using an item adapted from the European Community Respiratory Health Survey [31].

### Analysis

We began by tabulating the work history and current employment status of those with any form of respiratory disease, those with selected non-respiratory conditions, and those with no chronic conditions. In the remainder of the paper, persons with sleep apnea and lung cancer in the absence of COPD, asthma, and rhinitis were excluded from the analysis due to small numbers. We tabulated the work history and current employment status of persons with COPD, asthma, and rhinitis and compare the foregoing groups to those with selected other chronic conditions or with none. We then compare the demographic, health, and work characteristics of the persons with COPD, asthma, and rhinitis to those with selected non-respiratory conditions, and with no chronic conditions, using chi-square tests for categoric variables and F tests for continuous variables. Subsequently, we used the Kaplan-Meier method to estimate the duration of work life after age 25 for each of the groups in the study. Since the analysis was done retrospectively, there was no right censorship due to loss-to-follow-up, but those respondents who continued to be employed as of the interview year or as of age 65 (the typical retirement age) were right censored for the discontinuation of work. In the Kaplan-Meier analyses, the Wilcoxon test is used to compare pairs of conditions for the time until cessation of work activities.

Finally, we used Cox proportional hazards regression to estimate the impact of demographic characteristics and work-related variables for the longest job held on the number of years until cessation of work, again treating as censored observations those still employed at the time of interview. Sequential models with an increasing number of covariates associated with employment outcomes in prior studies in the literature were tested [32]. In the first, we estimated the risk of leaving work associated with COPD, asthma, rhinitis, and selected non-respiratory chronic conditions (with persons without chronic conditions serving as the reference category) after adjusting only for age. Next we added other demographic characteristics to the model, and then the set of work characteristics were added to the model including the demographic characteristics. The adjustment for demographic characteristics and work-related variables permits estimation of the extent to which early cessation of work among persons with respiratory conditions occurs independently of other characteristics that may jeopardize employment, such as low levels of education, adverse working conditions, and having held a job in a declining sector of the economy.

The demographic characteristics included in the Cox regression analysis were age, gender, race/ethnicity (Hispanic, African-American, with white not of Hispanic origin as the referent), marital status (never versus ever married), and extent of formal education (less than high school, high school graduate, some college, college graduate, with post-graduate as the referent). The work characteristics were measured for the longest held job and included occupation categorized as professional and managerial; sales, administrative and technical support; and manual labor, operatives, and crafts workers (with service workers as the referent); industry dichotomized to goods-producing versus services; and the presence or absence of self-reported exposure to vapors, gases, dust, or fumes.

Because of the complex sampling for the study, we performed sensitivity analyses to ascertain whether the results differed by sampling frame (random digit dialing frame versus the two "hot spot" frames combined). The results of the Kaplan-Meier and Cox regression analyses did not differ significantly or substantially by sampling frame. The results of the sensitivity analyses, therefore, are not reported below. To evaluate the proportional hazards assumption, we included interaction terms for condition groups and time in the Cox model; there was no evidence that the proportional hazards assumption was violated.

## Results

Comparing the work history and current employment status of persons with the three discrete respiratory conditions under study and those with selected non-respiratory conditions or with no chronic conditions, we find no statistically significant differences among the disease groups in the proportion who had ever worked (Table 1). However, persons with each of the three respiratory conditions and those with non-respiratory conditions were significantly less likely than those without chronic conditions to be employed when interviewed or at age 64. Persons with COPD were significantly less likely to be employed than those with asthma or rhinitis (31 percent for COPD versus 40 and 45 percent for asthma and rhinitis, respectively). Those with asthma and rhinitis did not differ from those with non-respiratory conditions in the proportion employed.

**Table 1:** Work History and Employment Status, by Disease Group

	**Work History**		**Employment Status at Interview or Age 64**	
		
**Disease Group**	**Never**	**Ever**^**3**^	**Employed**	**Not Employed**^**4**^
**Any Respiratory Condition**^**1**^	54 (7%)	687 (93%)	253 (37%)	434 (63%)
**COPD**	32 (9%)	334 (91%)	105 (31%)	229 (69%)
**Asthma**	9 (7%)	115 (93%)	46 (40%)	69 (60%)
**Rhinitis**	9 (5%)	185 (95%)	84 (45%)	101 (56%)
**Non-respiratory Conditions**^**2**^	65 (10%)	597 (90%)	265 (44%)	332 (56%)
**No Chronic Conditions**	61 (10%)	541 (90%)	301 (56%)	240 (44%)
**Total**	176 (9%)	1772 (91%)	801 (45%)	971 (55%)

^1 ^Includes sleep apnea and lung cancer, in addition to COPD, asthma, and rhinitis.

^2 ^Non-respiratory conditions include arthritis, diabetes, congestive heart failure, and coronary artery disease or heart attack.

^3 ^There were no significant differences in work history by disease group.

^4 ^All disease groups had significantly (p < 0.05) lower employment rates at interview (or age 64) than the group with no chronic conditions. Additionally, the COPD group had significantly lower employment rates than the rhinitis and non-respiratory condition groups. There were no other significant differences by disease group.

In the remainder of the paper, we limit our analysis to the 1772 respondents (91 percent) with a work history, excluding those with sleep apnea (n = 46) or lung cancer (n = 7) for whom sample sizes were too small to permit reliable estimations. Table 2 compares the health, demographic, and work characteristics of the persons with COPD, asthma, rhinitis, non-respiratory chronic conditions, and no chronic conditions. Persons with COPD reported a significantly and substantially lower SF-12 physical component score, a slightly lower SF-12 mental component score, and, along with those with asthma, elevated levels of comorbidity. As expected, persons with COPD were more likely to be a former or current smoker. They were also much more likely to report less than a high school education. Reflecting the conjoint role of occupational factors and smoking in the etiology and progression of COPD [27], persons with this condition were substantially more likely to report exposure to vapors, gases, dust, or fumes. They were also more likely to report being in manual and service occupations and in goods-producing industries.

**Table 2:** Health Characteristics at Time of Interview, Demographic Characteristics, and Work Characteristics at Longest Held Job among Persons with Work History, by Disease Group

**Kind of Characteristic**	**Total**	**COPD**	**Asthma**	**Rhinitis**	**Non-Respiratory Chronic Conditions**^**1**^	**No Chronic Conditions**	**p-value**
	**n = 1772**	**n = 334**	**n = 115**	**n = 185**	**n = 597**	**n = 541**	

**Health Characteristics at Time of Interview**							
SF-12 Physical Component Score, mean (± sd)	45 (± 12)	36 (± 13)	41 (± 13)	46 (± 12)	44 (± 12)	52 (± 7)	p < 0.001
SF-12 Mental Component Score, mean (± sd)	54 (± 9)	50 (± 11)	52 (± 10)	53 (± 10)	55 (± 8)	56 (± 7)	p < 0.001
Comorbid conditions, mean (± sd)	0.8 (± 1.0)	1.3 (± 1.1)	1.0 (± 1.1)	0.8 (± 0.9)	0.4 (± 0.7)	----	p < 0.001
Smoking Status, n (%)							
Never Smoked	674 (38%)	60 (18%)	51 (44%)	86 (46%)	243 (41%)	234 (43%)	p < 0.001
Former Smoker	751 (42%)	165 (49%)	50 (44%)	72 (39%)	261 (44%)	203 (38%)	
Current Smoker	347 (20%)	109 (33%)	14 (12%)	27 (15%)	93 (15%)	104 (19%)	
**Demographic Characteristics**							
Age at Interview, mean (± sd)	64 (± 6)	64 (± 6)	63 (± 6)	63 (± 6)	65 (± 6)	63 (± 6)	p < 0.001
Male, n (%)	784 (44%)	127 (38%)	38 (33%)	76 (41%)	275 (46%)	268 (50%)	p < 0.001
Race/Ethnicity, n (%)							
White, not of Hispanic Origin, and Other	1585 (89%)	296 (89%)	106 (92%)	165 (89%)	521 (88%)	492 (91%)	p = 0.11*
Hispanic	104 (6%)	13 (4%)	6 (5%)	13 (7%)	42 (7%)	30 (6%)	
African-American	83 (5%)	25 (7%)	3 (3%)	7 (4%)	29 (5%)	19 (3%)	
Marital Status, n (%)							
Never Married	61 (3%)	13 (4%)	5 (4%)	3 (2%)	17 (3%)	23 (4%)	p = 0.40
Ever Married	1711(97%)	321 (96%)	110 (96%)	182 (98%)	58 (97%)	518 (96%)	
Education, n (%)							
< HS	220 (13%)	65 (19%)	13 (11%)	20 (11%)	76 (13%)	46 (9%)	p < 0.001
HS Grad	484 (27%)	106 (32%)	25 (22%)	27 (15%)	193 (32%)	133 (25%)	
Some College	567 (32%)	96 (29%)	36 (31%)	65 (35%)	189 (32%)	181 (33%)	
College Grad	271 (15%)	37 (11%)	14 (12%)	32 (17%)	79 (13%)	109 (20%)	
Post-Grad	230 (13%)	30 (9%)	27 (24%)	41 (22%)	60 (10%)	72 (13%)	
**Work Characteristics at Longest Held Job**							
Self-Reported Exposure to Vapors, Gases, Dust, or Fumes, n (%)	709 (40%)	183 (55%)	50 (43%)	67 (36%)	216(36%)	193 (36%)	p < 0.001
Occupation, n (%)							
Professional or Managerial	532 (30%)	81 (24%)	44 (38%)	79 (43%)	141 (24%)	187 (35%)	p < 0.001
Sales, Administrative, Technical Support	532 (30%)	95 (28%)	30 (26%)	54 (29%)	200 (33%)	153 (28%)	
Manual Labor, Operatives, Crafts	424 (24%)	92 (28%)	25 (22%)	32 (17%)	149 (25%)	126 (23%)	
Services	284 (16%)	66 (20%)	16 (14%)	20 (11%)	1107 (18%)	75 (14%)	
Industry, n (%)							
Goods-Producing	376 (21%)	89 (27%)	20 (17%)	36 (19%)	118 (20%)	113 (21%)	p = 0.09
Services	1396 (79%)	245 (73%)	95 (83%)	149 (81%)	479 (80%)	428 (79%)	

^1 ^Non-respiratory conditions include arthritis, diabetes, congestive heart failure, and coronary artery disease or heart attack.

**Figure 1:**
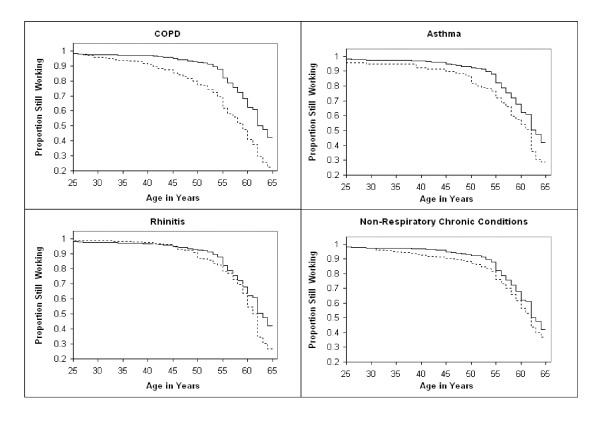
**Kaplan-Meier Estimates of Age until Cessation of Work Activities, by Disease Group**. Note: Solid line represents group with no chronic conditions; broken line represents the specified disease group. All conditions differ from group with no conditions (p < 0.05 by Wilcoxon Test). COPD differs from rhinitis group (p < 0.05 by Wilcoxon Test).

Figure 1 shows the results of the Kaplan-Meier estimates of the duration of work life after age 25 of persons with COPD, asthma, rhinitis and non-respiratory chronic conditions compared to that among persons with no chronic conditions. At age 35, there were only slight differences in the proportion still employed among the groups. At age 45, however, only 85 percent of persons with COPD were employed, while between 90 and 95 percent of the other groups were. At age 55, 62 percent of persons with COPD were still employed, while 72 and 78 percent of those with asthma and rhinitis, respectively were; at that age, 75 and 82 percent of those with selected non-respiratory chronic conditions and with no chronic conditions, respectively, were still employed. At age 64, just prior to the normal age of retirement, only 23 percent of those with COPD were still employed, as were 29 percent of those with asthma, 27 percent of those with rhinitis, 36 percent of those with selected non-respiratory chronic conditions, and 42 percent of those without chronic conditions. In formal tests of differences between pairs of groups in time until cessation of work, each of the respiratory condition groups as well as the non-respiratory condition group were found to leave work earlier than persons without chronic conditions (p < .05 by Wilcoxon test). In addition, persons with COPD left work earlier than those with rhinitis (p < .05), but in a comparison between persons with COPD and asthma, a test of the differences in time until cessation of work did not meet the traditional criterion for statistical significance (p = .06).

Table 3 displays the results of the Cox proportional hazards regressions. After adjustment for age differences among the groups, the hazard ratio for cessation of work associated with COPD was especially pronounced, 1.99 (95% CI 1.66 – 2.39), but the rates associated with asthma (1.48, 95% CI 1.13 – 1.94), rhinitis (1.34, 95% CI 1.06 – 1.69), and non-respiratory chronic disease (1.36, 95% CI 1.15 – 1.60) were also significantly elevated relative to persons without chronic conditions. The hazard ratios associated with COPD, asthma, rhinitis, and non-respiratory chronic disease were relatively unaffected by the addition of demographic and work characteristics, suggesting that the early cessation of work among persons in these groups was associated with the conditions themselves rather than their demographic backgrounds or the nature of their prior employment. In the Cox regression model including age, demographic characteristics, and work-related variables, each year of advancing age was associated with a significantly, albeit slightly decreased hazard for leaving work (hazard ratio of 0.95/year, 95% CI 0.94 – 0.96), as were those with less than a high school education (hazard ratio relative to those with some graduate school of 1.74, 95% CI 1.29, 2.35) and high school graduates (hazard ratio relative to those with some graduate school of 1.43, 95% CI 1.10 – 1.86), while women were significantly less likely to leave work in each year (hazard ratio of 0.79, 95% CI 0.69 – 0.91). No other demographic characteristic or work-related variable was significantly associated with the hazard of leaving work early.

**Table 3:** Cox Proportional Hazards Models for Years Until Cessation of Work Activities Until Age 65, among Persons with a Work History

	**Hazard Ratio (95% CI)**				
	
**Model**	**COPD**	**Asthma**	**Rhinitis**	**Non- Respiratory**	**No Chronic Conditions**
Disease Categories and Age	1.99 (1.66, 2.39)	1.48 (1.13, 1.94)	1.34 (1.06, 1.69)	1.33 (1.12, 1.57)	ref.
Disease Categories, Age, and Demographic Characteristics^1^	1.86 (1.55, 2.23)	1.46 (1.12, 1.92)	1.37 (1.08, 1.73)	1.28 (1.08, 1.52)	ref.
Disease Categories, Age, and Demographic and Work Characteristics^2^	1.83 (1.52, 2.21)	1.45 (1.11, 1.90)	1.37 (1.08, 1.73)	1.28 (1.08, 1.51)	ref.

^1 ^Demographic characteristics include gender, race/ethnicity, marital status, and extent of formal education

^2 ^Work characteristics include occupation, industry, and exposure to vapors, gases, dust, or fumes

## Discussion

Clinical and population-based studies of the employment outcomes of chronic diseases such as the respiratory conditions that are the subject of the present paper have competing advantages and disadvantages. In clinical studies, there is generally greater certainty of diagnosis and the opportunity to categorize disease history and disease severity with greater precision. With this precision, it is possible to provide individuals with more accurate information on their employment prognosis.

In population-based studies, there is an opportunity to establish the impact of conditions across a wide spectrum of disease. In addition to the assessment of work outcomes across a spectrum of disease, population-based studies often provide an opportunity to measure the impact of a condition or group of conditions against other major categories of disease and against a control group with no conditions when all groups have been sampled in the same way and have been interviewed at the same time and with the same battery of questionnaire items. This allows the investigator to assess the incremental impact of a marker condition or conditions, a much more rigorous way of measuring the effect of diseases on employment.

In the present study, we found that persons with COPD, asthma, and rhinitis do not differ from the remainder of the population of persons 55 to 75 in the proportion who have ever worked. However, among those with some work history, persons with COPD, asthma, and rhinitis were significantly less likely to report being currently employed when interviewed (or, among those past age 64, to have worked until that age) than those without chronic conditions.

Persons with COPD, asthma, rhinitis, and non-respiratory conditions all left work significantly earlier than those without chronic conditions, but the impact was especially pronounced among persons with COPD and the effect was particularly strong late in work life. The results of the Cox regression analyses corroborate the findings with respect to the extent of work life in showing that those with COPD experience a substantially higher hazard of early retirement, although persons in the other respiratory and the non-respiratory groups had significantly elevated hazards of early retirement relative to those without chronic conditions. The results from this national study with respect to overall employment rates are consistent with those from a population-based study among working age Californians [23] and in studies of those with non-respiratory clinical entities^21–22^, but extend those results to the analysis of the impact of chronic respiratory conditions on extent of work life.

The lower overall employment rate among persons with COPD (and, to a lesser extent, those with asthma and rhinitis) and their earlier withdrawal from the labor market may endanger their security in retirement since the level of pension benefits for most Americans is a function of the number of years worked. Also, the last few years of work are the time when much of one's retirement savings are accumulated, since prior to that point, many have significant expenses for childrearing and a home purchase [25]. Indeed, it is likely that persons with COPD are forced to "spend down" their assets faster than most older Americans due to their early retirement with ramifications for their own financial well-being, for that of their families, and to the extent they seek entitlement to such programs as Medicaid, for the society as a whole.

The results reported here may be affected by several limitations in the study design. Most prominently, the diagnoses of specific respiratory conditions were based on self-report of having received a physician's diagnosis of one of three conditions subsumed in the study definition of COPD (chronic bronchitis, emphysema, or chronic obstructive lung disease), asthma, or rhinitis. Specifically, we did not have pulmonary function data or results of CT scans. However, the prevalence of COPD is consistent with that from large-scale population-based studies, such as the National Health and Nutrition Examination Study.^27 ^Moreover, the study sample was similar in the distribution of such characteristics as gender, race/ethnicity, educational attainment, marital status, smoking status and history as other national data sources on persons 55 to 75.

Also, because this was a cross-sectional study of those living outside of institutions, it omitted many of those with the most severe forms of COPD who may reside in institutions. Obviously, those who died prior to the study's commencement may have left work before the normal retirement age, but were also omitted from the estimations. This may account for the finding that advanced age was significantly, although weakly associated with a decreased hazard of leaving work. These limitations probably resulted in offsetting biases. It is quite likely that persons with COPD who failed to receive a physician's diagnosis had relatively mild forms of the condition. If such persons had been included among those with a diagnosis of COPD, that would have resulted in lower rates of withdrawal from employment than we calculated. Inclusion of those residing in institutions, on the other hand, would likely have increased the calculated rate of withdrawal.

Another possible study limitation is that those who have left work may not report the age at which this occurred accurately. However, it should be pointed out that the estimated impact of COPD and the other respiratory conditions on duration of employment was consistent with the results for current employment status in showing the especially adverse impact of COPD on work, as well as the relatively smaller impact on persons with asthma, rhinitis, and non-respiratory conditions. Moreover, since the impact of COPD on withdrawal from work occurred relatively late in the potential work life of the respondents, recall bias is unlikely to substantially affect the estimates of the length of the work life.

## Conclusion

Overall, persons with COPD, asthma, and rhinitis are no less likely to have a work history than those with non-respiratory conditions or with none, but persons with COPD and, to a lesser extent, those with asthma and rhinitis, are much less likely to sustain their careers as long as those without chronic conditions.

## Competing interests

The author(s) declare that they have no competing interests.

## Authors' contributions

EY helped to design the survey instrumentation, designed the analyses, assisted in the data analysis, and wrote the manuscript. PK helped to design the survey instrumentation, assisted in the design of the analyses, and reviewed the manuscript. JB assisted in the design of the analyses, provided input as to the respiratory conditions under study, and reviewed the manuscript. LT wrote the analysis plan with EY, performed some of the analyses, and assisted in the composition of the manuscript. GE performed the majority of the analyses and assisted in the preparation of the manuscript. ME helped to obtain the research support, assisted in the interpretation of the literature about the respiratory conditions under study, reviewed the analyses, and assisted in the preparation of the manuscript. PB was the principal investigator on the research grant that supported the study, helped to design the survey instrumentation, provided clinical input in the design of the analyses, reviewed the analyses, and assisted in the preparation of the manuscript.

Grant Support: HL677438; HL04201 (National Heart Lung and Blood Institute)

## References

[B1] McClellan VE, Garrett JE (1990). Asthma and the employment experience. N Z Med J.

[B2] Sibbald B, Anderson HR, McGuigan S (1992). Asthma and employment in young adults. Thorax.

[B3] Gannon PF, Weir DC, Robertson AS, Burge PS (1993). Health, employment, and financial outcomes in workers with occupational asthma. Br J Ind Med.

[B4] Blanc P, Jones M, Besson C, Katz P, Yelin E (1993). Work disability among adults with asthma. Chest.

[B5] Ameille J, Pairon JC, Bayeux MC, Brochard P, Choudat D, Conso F, Devienne A, Garnier R, Iwatsubo Y (1997). Consequences of occupational asthma on employment and financial status: a follow-up study. Eur Resp J.

[B6] Ross SG, Rupp K, Stapleton DC (1998). The Perspective of a Public Trustee. Growth in Disability Benefits.

[B7] Balder B, Lindholm NB, Lowhagen O, Palmqvist M, Plaschke P, Tunsater A, Toren K (1998). Predictors of self-assessed work ability among subjects with recent-onset asthma. Respir Med.

[B8] Gassert TH, Hu H, Kelsey KT, Christiani DC (1998). Long-term health and employment outcomes of occupational asthma and their determinants. J Occup Environ Med.

[B9] Yelin EH, Henke J, Katz P, Eisner M, Blanc P (1999). The work dynamics of adults with asthma. Amer J Indus Med.

[B10] Blanc PD, Ellbjar S, Janson C, Norback D, Norrman E, Plaschke P, Toren K (1999). Asthma-related work disability in Sweden. The impact of workplace exposures. Am J Respir Crit Care Med.

[B11] Tarlo SM, Leung K, Broder I, Silverman F, Holness DL (2000). Asthmatic subjects symptomatically worse at work: prevalence and characterization among general asthma clinic population. Chest.

[B12] Diette GB, Markson L, Skinner EA, Nguyen TT, Algatt-Bergstrom P, Wu AW (2000). Nocturnal asthma in children affects school attendance, school performance, and parents' work attendance. Arch Pediatr Adolesc Med.

[B13] Ungar WJ, Coyte PC (2000). Measuring productivity loss days in asthma patients. The Pharmacy Medication Monitoring Program and Advisory Board. Health Econ.

[B14] Blanc P, Trupin L, Eisner M, Earnest G, Katz P, Israel L, Yelin E (2001). The work impact of asthma and rhinitis: Findings from a population-based survey. J Clin Epidemiol.

[B15] Larbanois A, Jamart J, Delwiche JP, Vandenplas O (2002). Socioeconomic outcome of subjects experiencing asthma symptoms at work. Eur Respir J.

[B16] Smith LA, Hatcher JL, Wertheimer R (2002). The association of childhood asthma with parental employment and welfare report. JAMWA.

[B17] Blanc PD, Burney P, Janson C, Toren K (2003). The prevalence and predictors of respiratory-related work limitation and occupational disability in an international study. Chest.

[B18] Saarinen K, Karjalainen A, Martikainen R, Uitti J, Tammilehto L, Klaukka T, Kurppa K (2003). Prevalence of work-aggravated symptoms in clinically established asthma. Eur Resp J.

[B19] Crystal-Peters J, Crown WH, Goetzel RZ, Schutt DC (2000). The cost of productivity losses associated with allergic rhinitis. Am J Manag Care.

[B20] Gillen M, Lallas D, Brown C, Yelin E, Blanc P (1998). Work disability in adults with cystic fibrosis. Am J Respir Crit Care Med.

[B21] Yelin EH (1995). Musculoskeletal conditions and employment. Arthritis Care Res.

[B22] Kessler RC, Greenberg PE, Mickelson KD, Meneades LM, Wang PS (2001). The effects of chronic medical conditions on work loss and work cutback. J Occup Environ Med.

[B23] Eisner MD, Yelin EH, Trupin L, Blanc PD (2002). The influence of chronic respiratory conditions on health status and work disability. Am J Pub Health.

[B24] Ward MM, Javitz HS, Smith WM, Whan MA (2002). Lost income and work limitations in persons with chronic respiratory disorders. J Clin Epi.

[B25] Haveman R, Budetti PP, Burkhauser RV, Gregory JM, Hunt HA (2001). Social Insurance and the Older Worker. Ensuring Health and Income Security for an Aging Workforce.

[B26] Nichols LM, Budetti PP, Burkhauser RV, Gregory JM, Hunt HA (2001). Policy Options for Filling Gaps in the Health Insurance Coverage of Older Workers and Early Retirees. Ensuring Health and Income Security for an Aging Workforce.

[B27] Trupin L, Earnest G, San Pedro M, Balmes JR, Eisner M, Yelin E, Katz P, Blanc P (2003). The occupational burden of chronic obstructive pulmonary disease. Eur Respir J.

[B28] Ware J, Kosinski M, Keller S (1996). A 12-item short form health survey: Construction of scales and preliminary tests. Medical Care.

[B29] U.S. Bureau of the Census (1993). Current Population Survey Technical Documentation.

[B30] U.S. Bureau of the Census (2000). Industry and Occupation Classification System.

[B31] United Medical and Dental Schools of Guy's and St. Thomas' Hospitals, Department of Public Health Medicine (1993). Protocol for the European Community Respiratory Health Survey.

[B32] Yelin EH (1992). Disability and the Displaced Worker.

